# Etiologies of Extreme Leukocytosis

**DOI:** 10.7759/cureus.38062

**Published:** 2023-04-24

**Authors:** Esther Viner, Judith Berger, Victoria Bengualid

**Affiliations:** 1 Internal Medicine, City University of New York (CUNY) School of Medicine, New York City, USA; 2 Infectious Diseases, St. Barnabas Hospital (SBH) Health System, New York City, USA

**Keywords:** patient etiology, site of infection, reactive leukocytosis, infection rates, leukocytosis

## Abstract

Objective: The objective of this study was to determine the etiologies and co-morbidities associated with extreme leukocytosis, which is characterized by a white blood cell (WBC) count ≥ 35 × 10^9 ^leukocytes/L.

Method: Retrospective chart review was conducted for all patients, aged 18 years and older, admitted to the internal medicine department between 2015 and 2021 with an elevated WBC count ≥ 35 × 10^9^ leukocytes/L within the first 24 hours of admission.

Results: Eighty patients were identified to have WBC count ≥ 35 × 10^9^ leukocytes/L. The overall mortality was 16% and increased to 30% in those presenting with shock. Mortality increased from 2.8% in patients with WBC count in the range of 35-39.9 × 10^9^ leukocytes/L to 33% in those with WBC count in the range of 40-50 × 10^9^ leukocytes/L. There was no correlation with underlying co-morbidities or age. Pneumonia was the most common infection (38%), followed by UTI or pyelonephritis (28%) and abscesses (10%). There was no predominant organism responsible for these infections. The most common etiology for WBC count between 35-39.9 × 10^9^ leukocytes/L and 40-50 × 10^9^ leukocytes/L was infections, while malignancies (especially chronic lymphocytic leukemia) were more common with WBC count > 50 × 10^9^ leukocytes/L.

Conclusion: For WBC counts in the range of 35-50 × 10^9^ leukocytes/L, infections were the main reason for admission to the internal medicine department. Mortality increased from 2.8% to 33% as WBC counts increased from 35-39.9 × 10^9 ^leukocytes/L to 40-50 × 10^9 ^leukocytes/L. Overall, mortality for all WBC counts ≥ 35 × 10^9^ leukocytes/L was 16%. The most common infections were pneumonia, followed by UTI or pyelonephritis and abscesses. The underlying risk factors did not correlate with WBC counts or mortality.

## Introduction

Leukocytosis refers to a white blood cell (WBC) count of more than 11 × 10^9^ leukocytes/L. The etiologies of elevated WBC count encompass infections, malignancies, hemorrhages, hemolysis, intoxication, and other entities that cause severe stress [1]. The definition of moderate, severe, and extreme leukocytosis varies from article to article. For example, Hasjim et al. examined moderate to severe leukocytosis in trauma patients [1]. They defined moderate leukocytosis as WBC count in the range of 25-40 × 10^9 ^leukocytes/L, and they considered any value greater than 40 × 10^9^ leukocytes/L as indicative of severe leukocytosis. Granger et al. examined the etiologies and outcomes of extreme leukocytosis in patients with solid tumors [2]. They recruited patients with a WBC count of 40 × 10^9^ leukocytes/L. A third study enrolled 100 patients with extreme leukocytosis with a WBC count > 25 × 10^9^ leukocytes/L [3]. We chose to study patients with a WBC count ≥ 35 × 10^9^ leukocytes/L on admission to the internal medicine department, to determine the etiologies and outcomes for these patients with extreme leukocytosis.

## Materials and methods

This study was conducted in St. Barnabas Hospital Health System, a community-based not-for-profit 400-bed acute care and level 2 trauma center hospital, located in the Bronx, New York, United States. The study was approved by St. Barnabas Hospital's Institutional Review Board in 2021 (approval number: number 27).

Using electronic medical records, we retrospectively reviewed the records of all patients, aged 18 years and older, who were admitted to the internal medicine department from 2015 to 2021 with a WBC count ≥ 35 × 10^9^ leukocytes/L. Patients were included if they were admitted to the internal medicine department and had at least one WBC reading ≥ 35 × 10^9^ leukocytes/L within 24 hours of admission. The charts were analyzed for demographical information, diagnoses, culture results, and underlying risk factors (diabetes, hypertension, hyperlipidemia, chronic renal disease, asthma, chronic obstructive pulmonary disease, human immunodeficiency virus-1, bedbound status, cerebral vascular accident, alcohol use, and coronary heart disease). 

Etiology was defined as infectious if the patient had a positive culture from a sterile site, the chest radiograph was suggestive of pneumonia, there was evidence of skin or soft tissue infection, or there was clinical improvement in response to antibiotic treatment. Admission was attributed to malignancy or complication from chemotherapy in patients without an infectious or other etiology. This included patients with newly diagnosed malignancies, patients admitted with tumor lysis syndrome, and patients with dehydration on Neupogen. All other etiologies were categorized as “other.”

## Results

Eighty patients were identified to have WBC counts in the range 35-168.5 × 10^9^ leukocytes/L. Fifty-one percent were male. This study spanned the years 2015-2021, with an average of one case of extreme leukocytosis per month. The median age was 61.5 years, with a range of 21 years to 95 years. Co-factors such as age, diabetes, cardiac risk factors, hypertension, chronic kidney disease, alcohol use, and bedbound status did not correlate with mortality or degree of WBC count elevation (Table 1).

**Table 1:** Mortality and underlying risk factors in patients grouped according to WBC count values. COPD: chronic obstructive pulmonary disease; HIV: human immunodeficiency virus; CAD: coronary artery disease; CHF: congestive heart failure; CVA: cerebral vascular accident. None of the risk factors showed statistical significance.

WBC: leukocytes/L	35-39.9 × 10^9^	40-50 × 10^9^	> 50 × 10^9^
N	36	27	17
Age average (range)	61 (21-95)	65 (22-92)	51 (24-84)
Male	19 (53%)	13 (48%)	9 (53%)
Diabetes	12 (33%)	10 (37%)	3 (18%)
Hypertension	15 (42%)	15 (55%)	6 (35%)
Hyperlipidemia	5 (14%)	4 (15%)	1 (5.9%)
Chronic renal disease	3 (8.3%)	2 (7.4%)	1 (5.9%)
COPD/asthma	6 (17%)	2 (7.4%)	2 (11.8%)
Alcohol use	2 (5.5%)	2 (7.4%)	2 (11.8%)
HIV	3 (8.3%)	1 (3.7%)	0
Bedbound status	6 (17%)	4 (15%)	1 (5.9%)
CAD/CHF	3 (8.3%)	1 (3.7%)	0
CVA	1 (2.8%)	2 (7.4%)	1 (5.9%)

Etiologies of extreme leukocytosis were categorized as resulting from infections (n=50), malignancies (n=20), and other reasons (n=10), which included three patients with COPD or asthma exacerbation, two patients with trauma (stabbing and intracranial hemorrhage), two patients with cardiac arrest, one patient with diabetic ketoacidosis, one patient in cardiogenic shock, and one patient with variceal bleeding. Overall mortality was 16% (13 patients). The mortality was higher (28%) in patients who presented with shock (n=25). An infectious etiology was responsible for nine of the 13 patients who died. 

**Figure 1:**
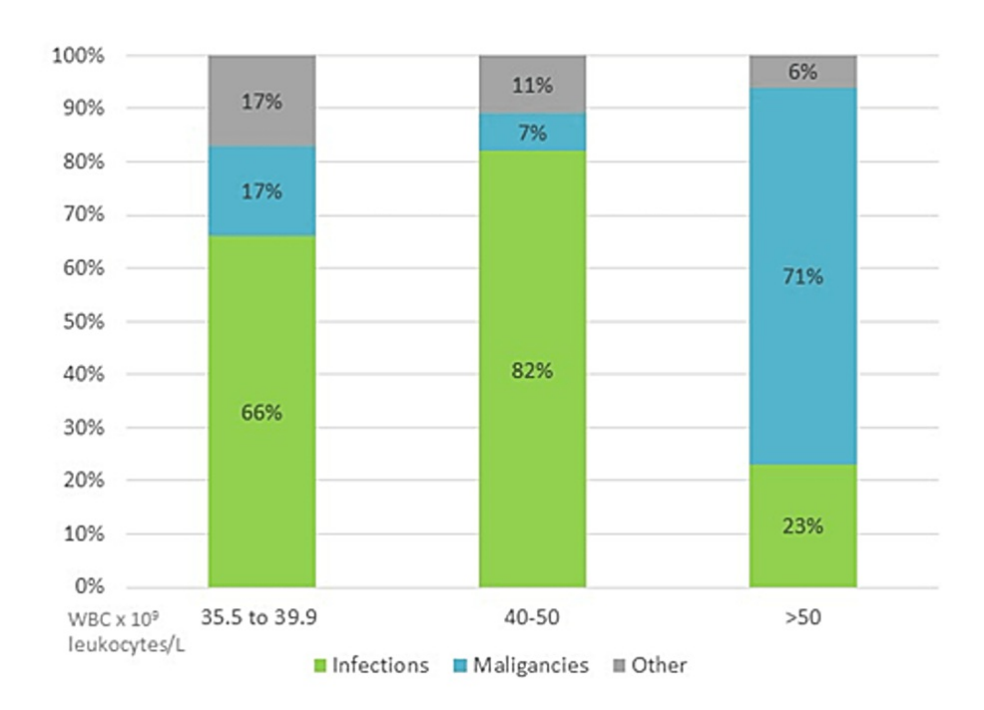
Etiologies of extreme leukocytosis. Patients grouped by WBC count values: 35-39.9 × 10^9^ leukocytes/L, 40-50 × 10^9^ leukocytes/L, and greater than 50 × 10^9^ leukocytes/L.

Patients were divided into three groups by WBC counts: 35-39.9 × 10^9^ leukocytes/L, 40-50 × 10^9^ leukocytosis/L, and greater than 50 × 10^9^ leukocytes/L. Figure 1 graphs the etiologies of elevated WBC counts in these three groups of patients. Tables 2, 3 summarize the infectious and malignant etiologies for the elevated WBC counts. Mortality increased from 2.8% in the group with WBC count in the range of 35-39.9 × 10^9^ leukocytes/L (n=36) to 33% in patients admitted with WBC count in the range of 40-50 × 10^9^ leukocytes/L (n=27). In the group with WBC counts > 50 × 10^9^ leukocytes/L (n=17), mortality decreased to 18%, as this group mainly included patients with malignancies.

**Table 2:** Infectious etiologies including pathogens in patients admitted with extreme leukocytosis. MRSA: methicillin-resistant *Staphylococcus aureus; *COVID-19: coronavirus disease 2019; MSSA: methicillin-sensitive *Staphylococcus aureus*

Types of infections	N (%)	Pathogens
Pneumonia	19 (38%)	*Pseudomonas aeruginosa*, *Staphylococcus aureus*, *Streptococcus pneumonia*, COVID-19, *Acinetobacter*, *Kocuria kristinae*, *Peptostreptococcus*, *Streptococcus viridans*, empyema
UTI or pyelonephritis	14 (28%)	*Escherichia coli*, *Enterococcus*, *Pseudomonas*; *Klebsiella pneumonia*; *Citrobacter, Pseudomonas* *aeruginosa,* *Morganella morgani*
Abscesses	5 (10%)	*Streptococcus gallolyticus* (*pasteurianus*) (pancreatic), *E. coli *(pelvic), group A *Streptococcus* (neck), MRSA (periorbital); MSSA (gluteal)
Cellulitis	4 (8%)	*Pseudomonas aeruginosa*, *Staphylococcus epidermidis* (prosthetic device), group A *Streptococcus*
Cholecystitis or cholangitis	3 (6%)	E. coli
*Clostridium difficile* colitis	3 (6%)	
Peripherally inserted central catheter (PICC) or midline catheter	3 (6%)	S. epidermidis, Acinetobacter
Necrotizing fasciitis	1 (2%)	Bacteroides, *E. coli*, *Enterococus faecalis*
Fasciitis	1 (2%)	Group A *Streptococcus*
Sacral decubitis	1 (2%)	*E. faecalis* and *E. coli*
Hip Infection	1 (2%)	MS*SA*
Diverticulitis with abscess	1 (2%)	Peptostreptococcus
Cryptococcal meningitis	1 (2%)	Chronic lymphocytic leukemia (HIV negative)

**Table 3:** Malignancies by level of leukocytosis. CLL: chronic lymphocytic leukemia, CML: chronic myeloid leukemia, ATLL: adult T-cell leukemia/lymphoma, ALL: acute lymphocytic leukemia, AML: acute myeloid leukemia

Malignancies	35-39.9 × 10^9 ^leukocytes/L	40-50 × 10^9 ^leukocytes/L	>50 × 10^9 ^leukocytes/L
Solid tumors	Sarcoma, breast, colon	Breast, cervical	Uterine, adenocarcinoma, breast
Leukemia	ATLL, CML	None	CLL, ALL, AML
Underlying malignancies but not the cause of admission	CLL, cervical cancer-causing urinary obstruction	CLL with pneumonia	CML with COVID-19 pneumonia, plasma cell with fasciitis, breast cancer with cellulitis

Focusing on etiologies for admission in the group of patients with the highest WBC count (> 50,000 × 10^9^ leukocytosis/L) (n=17), 71% were due to a malignancy (mainly chronic lymphocytic leukemia (CLL)) or a complication from a malignancy (dehydration, Neupogen use, and transfusion-related acute lung injury after a blood transfusion). Twenty-three percent were due to infection: a nursing home patient with sepsis due to an infected sacral ulcer with positive blood cultures for *Enterococcus faecium *and *Escherichia* *coli*, a patient with breast cancer and breast reconstruction with expanders admitted with breast cellulitis infected with *Pseudomonas *and *Staphylococcus epidermidis*, a patient with chronic myeloid leukemia (CML) admitted with pneumonia caused by coronavirus disease 2019 (COVID-19), and one patient with underlying plasma cell cancer admitted with fasciitis with positive blood cultures for group A *Streptococcus*. One patient in the “other” category had intracranial bleeding secondary to trauma.

## Discussion

A review of the literature yielded several articles examining specific aspects of leukocytosis. For example, *Clostridioides difficile *(formerly known as *Clostridium difficile*), has been associated with elevated WBC counts. In their study, Teja et al. wanted to determine if the combination of extreme leukocytosis (defined as WBC count ≥ 30 × 10^9^ leukocytes/L) and diarrhea was predictive of *C. difficile *infection [4]. In these patients with an elevated WBC count and diarrhea, 15% had *C. difficile *infection with a mortality rate of 33.8%. In our study, *C. difficile *colitis was the etiology in three patients with WBC counts of 38.1, 38.6, and 43.1 × 10^9^ leukocytes/L. One patient died during their admission.

Another study examined severe leukocytosis in trauma patients [1]. The authors reviewed 15,807 trauma patients. In the study, 2.1% of patients had moderate (WBC count in the range of 25-39 × 10^9^ leukocytes/L) or severe leukocytosis (WBC count ≥ 40 × 10^9^ leukocytes/L). The mortality rate was higher in the severe leukocytosis group (29.9% vs. 13.3%). However, the authors believed that this was due to vasopressor use, as it was the strongest predictor of mortality.

A third study focused on the etiologies and outcomes of extreme leukocytosis in patients with solid tumors [2]. The authors defined extreme leukocytosis as ≥ 40 × 10^9^ leukocytes/L. Of the 758 patients with solid tumors, 20% had extreme leukocytosis. Only 15% of the patients had an infectious etiology. The most common etiologies for elevated WBC count were hematopoietic growth factors (69%), high-dose steroids (5%), and newly diagnosed leukemia (1%).

In a prospective study of hospitalized patients with a leukemoid reaction (defined as a WBC count > 30 × 10^9^ leukocytes/L), in patients without hematologic malignancies, infections were the most common etiology (70%), followed by malignancy (7.5%) and bleeding (6.3%) [5]. The mortality rate in these patients was 34%. In this study, the authors tried to determine if any peripheral smear finding was helpful in predicting prognosis. They found that neutrophil vacuolation was associated with infection (34%), but this was a very labor-intensive process and, therefore, of minimal clinical value. In a similar but retrospective study by Potasman et al., WBC count ≥ 30 × 10^9^ leukocytes/L in patients without malignancies was due to infections (48%), followed by ischemia or stress (27.7%), inflammation (6.9%), and obstetric diagnoses (6.9%) [6]. The overall mortality rate was 38.1%, and age as well as any infectious diagnosis highly correlated with death.

Finally, there are other studies that examined leukocytosis in the setting of Veterans Affairs and in the emergency room. In a study published in 1998, the authors followed 100 patients presenting to their Veterans Affairs Medical Center from 1993 to 1994 with a WBC count ≥ 25 × 10^9^ leukocytes/L with more than 50% granulocytes [3]. The authors found that infections were associated with 45% of the cases. Another article, published in 2007 by Lawrence et al., compared patients presenting to the emergency room with a WBC count > 25 × 10^9^ leukocytes/L to those with a WBC count in the range of 12-25 × 10^9^ leukocytes/L [7]. Patients with extreme leukocytosis had more infections (74% vs. 48%) and were more likely to die (32.1% vs. 12.7%).

In our study, infections accounted for 62.5% of all admissions, followed by malignancies (25%). The overall mortality rate was 16%, which is lower than that in previous studies. The mortality rate increased to 18% in patients presenting with infections. The highest mortality rate (30%) occurred in patients who presented with septic shock. The mortality rate increased from 2.8% in the group of patients who presented with WBC counts in the range of 35.5-39.9 × 10^9^ leukocytes/L to 33% in those with WBC counts in the range of 40-50 × 10^9^ leukocytes/L.

Previous studies did not focus on the causes of infections. In our study, the most common infectious etiologies accounting for 75% of the presentations were pneumonia, followed by UTI or pyelonephritis and abscesses. Many different pathogens were identified as causing infections without a predominant type or correlation with mortality. The limitations of our study include its retrospective nature, the relatively small number of patients in each group, the consideration of admissions only to the internal medicine department, thereby excluding some surgical and obstetric patients, and the restriction of data to one inner-city hospital.

## Conclusions

Our retrospective chart review from 2015-2021 of patients with extreme leukocytosis admitted to the internal medicine department of an inner-city community hospital found that infections were responsible for the majority of the cases when WBC counts were in the range of 35-50 × 10^9^ leukocytes/L, while malignancies were seen more commonly with WBC counts > 50 × 10^9^ leukocytes/L. The most common infectious etiology was pneumonia, followed by UTI or pyelonephritis and abscesses. Underlying risk factors or pathogens did not correlate with WBC counts or mortality. Overall mortality was 16%. However, the mortality did change within the different WBC subgroups: 2.8% for WBC count in the range of 35-39.9 × 10^9 ^leukocytes/L (66% due to infections, 17% malignancies), 33% for WBC count in the range of 39.9-50 × 10^9^ leukocytosis/L (82% due to infections, 7% malignancies), and 18% for WBC count > 50 × 10^9 ^leukocytes/L (23% due to infections, 71% malignancies). 
